# Preparation of Exosomes for siRNA Delivery to Cancer Cells

**DOI:** 10.3791/58814

**Published:** 2018-12-05

**Authors:** Farid N. Faruqu, Lizhou Xu, Khuloud T. Al-Jamal

**Affiliations:** 1 Institute of Pharmaceutical Science, King's College London

**Keywords:** Cancer Research, Issue 142, Exosome, Isolation, Characterization, siRNA Delivery, Cellular Uptake, Nanocarrier

## Abstract

Extracellular vesicles, in particular exosomes, have recently gained interest as novel drug delivery vectors due to their biological origin, abundance, and intrinsic capability in intercellular delivery of various biomolecules. This work establishes an isolation protocol to achieve high yield and high purity of exosomes for siRNA delivery. Human Embryonic Kidney cells (HEK-293 cells) are cultured in bioreactor flasks and the culture supernatant (hereon referred to as conditioned medium) is harvested on a weekly basis to allow for enrichment of HEK-293 exosomes. The conditioned medium (CM) is pre-cleared of dead cells and cellular debris by differential centrifugation and is subjected to ultracentrifugation onto a sucrose cushion followed by a washing step, to collect the exosomes. Isolated HEK-293 exosomes are characterized for yield, morphology and exosomal marker expression by nanoparticle tracking analysis, protein quantification, electron microscopy and flow cytometry, respectively. Small interfering RNA (siRNA), fluorescently labeled with Atto655, is loaded into exosomes by electroporation and excess siRNA is removed by gel filtration. Cell uptake in PANC-1 cancer cells, after 24 h incubation at 37 °C, is confirmed by flow cytometry. HEK-293 exosomes are 107.0 ± 8.2 nm in diameter. The exosome yield and particle-to-protein ratio (P:P) ratio are 6.99 ± 0.22 × 10^12^ particle/mL and 8.3 ± 1.7 × 10^10^ particle/µg, respectively. The encapsulation efficiency of siRNA in exosomes is ~ 10-20%. Forty percent of the cells show positive signals for Atto655 at 24 h post-incubation. In conclusion, exosome isolation by ultracentrifugation onto sucrose cushion offers a combination of good yield and purity. siRNA could be successfully loaded into exosomes by electroporation and subsequently delivered into cancer cells *in vitro.* This protocol offers a standard procedure for developing siRNA-loaded exosomes for efficient delivery to cancer cells.

## Introduction

Exosomes are a subtype of extracellular vesicles (EV) ranging from 50-200 nm in diameter, secreted by various cell types such as immune cells^1^,^2^, cancer cells^3^,^4^,^5^,^6^ and stem cells^7^. Exosomes have also been shown to be present in various physiological fluids^8^,^9^,^10^,^11^. The combination of the inherent ability of exosomes to carry various biomolecules (*e.g.,* RNA and proteins)^12^,^13^,^14^ and the effective delivery of these biomolecules into recipient cells^15^,^16^,^17^ attracted interest for their potential as nano-scale drug delivery vectors. Various small molecules that serve as anti-cancer and anti-inflammatory drugs have been demonstrated to be successfully loaded into exosomes and delivered to target cells^18^,^19^,^20^,^21^,^22^,^23^,^24^,^25^,^26^,^27^. Interestingly, nucleic acids such as siRNA^28^,^29^ and microRNA^30^ have also been successfully loaded into exosomes *via* electroporation and delivered to target cells.

Recently, RNA interference (RNAi) *via* small interfering RNA (siRNA) has gained more interest as the preferred mechanism in gene silencing due to its high specificity, potent effect, minimal side effects and ease of siRNA synthesis^28^,^29^. siRNAs are double-stranded RNA molecules ranging from 19 to 25 nucleotides in length that triggers sequence-specific catalytic mRNA knockdown. Due to its large molecular weight and polyanionic nature, passive uptake of naked siRNA into cells is hindered^28^,^29^. It is also not possible for naked siRNA to be injected into the systemic circulation due to rapid degradation by plasma nucleases^31^. Thus, encapsulation of siRNA in a nanocarrier would aid the effective delivery and uptake of siRNA into the target cells.

Exosomes are an ideal system for siRNA encapsulation as its structure is comprised of a hollow, aqueous core enveloped by a phospholipid bilayer. Exosomes not only have good stability in the blood but also have natural targeting properties to deliver functional RNA into cells^32^. The study conducted by Alvarez-Erviti *et al.* successfully demonstrated effective delivery of siRNA to the brains of mice using engineered exosomes with virtually no complications^31^. It is hypothesized that exosome-based therapy is relatively safer than other therapies as exosomes do not replicate endogenously as cells would and therefore do not exhibit metastatic properties^15^.

Various methods have been reported to successfully isolate exosomes from either cell culture or physiological fluids. The most popular method uses ultracentrifugation to pellet exosomes from the starting material^31^,^32^,^33^. This method can be quite harsh on exosomes and usually co-precipitates proteins from the sample. Combining ultracentrifugation with a density-based separation such as sucrose gradients is becoming more common, to reduce protein and non-exosomal contamination in the isolated exosomes^19^,^34^. Size-exclusion chromatography (SEC) allows separation of exosomes from other types of extracellular vesicles (EV) by size and can also result in minimal protein contamination but is limited by small amount of starting material it can process^35^,^36^. Immunoaffinity capture uses beads coated with antibodies that bind to exosomal surface proteins such as tetraspanins or other cell-specific marker that allows specific capture of exosomes rather than EVs or other proteins, as well as isolating sub-population of exosomes from whole samples, but again is limited by the amount of starting material and is costly^36^,^37^. Polymer-based precipitation of exosomes used to be popular too, but since it is a rather crude precipitation, it leads to a higher non-exosomal vesicle and protein contamination^38^,^39^.

Electroporation has been reported for its inefficiency as a method to load exosomes with siRNA due to protein aggregation^15^,^28^,^31^. Transfection-based approaches were demonstrated to have better loading efficiency and protein stability, but is undesirable due to its toxicity and side effects of transfection agents in altering cellular gene expression^28^. Thus, electroporation has been more widely used in siRNA loading into exosomes as it is a safer method. However, an optimized encapsulation method needs to be established in order to deliver adequate amounts of siRNA to the target site for a potent gene knockdown.

Here, we propose an exosome isolation protocol using density-based ultracentrifugation onto just a single 25% (w/w) sucrose cushion prepared in deuterium oxide, rather than a sucrose density gradient. This is a cost-effective method that circumvents the laborious density gradient preparation and allows processing of large volumes of starting material, yet results in intact exosomes of high yield and purity suitable for subsequent loading with siRNA. Fluorescent Atto655-conjugated non-specific siRNA was loaded into Human Embryonic Kidney cells (HEK-293 cells) derived exosomes *via* electroporation and delivered to human pancreatic adenocarcinoma (PANC-1) cancer cells *in vitro.*

## Protocol

### 1 Cell Culture in a Bioreactor Flask

[Fig F1]

1. Culture HEK-293 cells in **normal medium** (see **Table of Materials;** 5% CO_2_, 37 °C) and expand them into 4 x T75 flasks (until 90% confluent).
2. Wet the membrane of the bioreactor flask by adding 50-100 mL of normal medium in the medium reservoir of the bioreactor flask.
3. Collect all HEK-293 cells from the 4 x T75 and resuspend them in 15 mL of **exosome-depleted medium** (see **Table of Materials**).
4. Add the HEK-293 cell suspension to the cell compartment of the bioreactor flask using a 20 mL syringe connected to a blunt fill needle (see **Table of Materials**), with care to remove any bubble that might have formed.
5. Fill the medium reservoir of the bioreactor flask with **normal medium** up to 500 mL and keep the flask in the incubator (5% CO_2_, 37 °C) for a week.

### 2 Conditioned Medium (CM) Harvesting from the Bioreactor Flask

1. After 1 week, discard all the medium in the medium reservoir of the bioreactor flask.
2. Remove all the medium in the cell compartment (*i.e.,* the **CM**) using a 20 mL syringe connected to a blunt fill needle.
3. Add 50-100 mL of **normal medium** to the medium reservoir.
4. Add 15 mL of **exosome-depleted medium** to the cell compartment by removing the old medium and adding fresh **exosome-depleted medium** using a 20 mL syringe connected to a blunt fill needle.
5. Fill the medium reservoir of the bioreactor flask with **normal medium** up to 500 mL and keep the flask in the incubator (5% CO_2_, 37 °C) for another week.
  NOTE: The culture can be continued for more than a year. For step 2.2, the CM from the first harvest will not be used for exosome isolation and is discarded. For the 2^nd^ and subsequent harvest, the CM is kept for exosome isolation.

### 3 Exosome Isolation onto a Sucrose Cushion

[Fig F2]

1. Pre-clear the CM (from step 2.2) by differential centrifugation and filtration as follows.
  1. Centrifuge at 500 x g for 5 min at 4 °C. Transfer the supernatant into a new tube and discard the pellet. Repeat this centrifugation step once more, recovering the supernatant and discarding the pellet.
  2. Centrifuge the supernatant from step 3.1.1 at 2,000 x g for 15 min and 4 °C and then discard pellet. Filter the recovered supernatant once through 0.22 µm filters.
2. During pre-clearing, prepare 25% (w/w) sucrose solution in deuterium oxide by accurately weighing out 1.9 g (± 0.001 g) of sucrose in a universal tube, and then topping up with deuterium oxide until the weight reaches 7.6 g (± 0.001 g).
3. Fill up an ultracentrifuge tube (see **Table of Materials**) with 22.5 mL of pre-cleared CM. Make up the CM to 22.5 mL with 0.22 µm-filtered PBS if the current volume is less than that.
4. Place a glass pipette (see **Table of Materials**) in the tube and, through it, add 3 mL of sucrose solution so that the solution forms a separate layer beneath the CM.
5. Carefully place the tube containing layered CM/sucrose solution into the bucket of a swing-out rotor (see **Table of Materials**), and secure the bucket into the rotor.
6. Place the rotor into the ultracentrifuge (see **Table of Materials**) and spin at 100,000 x g for 1.5 h at 4 °C.
7. Collect 2 mL of the sucrose layer and add this to an ultracentrifuge bottle (see **Table of Materials**) containing 20 mL of filtered PBS for a washing step.
8. Place the tubes into a fixed-angle rotor (see **Table of Materials**) and spin at 100,000 x g for 1.5 h at 4°C.
9. Carefully remove the supernatant with a 10 mL serological pipette and resuspend the pellet with 400 µL filtered PBS. Keep this exosome stock at 4 °C or -80 °C for short-term and long-term storage respectively.

### 4 Characterization of Exosome Size and Yield by Nanoparticle Tracking Analysis (NTA)

1. Make 1:1,000-1:50,000 dilutions of the exosome stock in 1 mL (minimum 750 µL) volume so as to obtain 20-80 particles in the viewing frame of the NTA instrument (see **Table of Materials**) display.
2. Inject the diluted exosome stock into the NTA instrument sample chamber using a 1 mL syringe, and insert the temperature probe of a thermometer into the temperature probe inlet.
3. Set the NTA software (see **Table of Materials**) for recording as follows: 3 standard measurements, 30 s each, manual temperature option unchecked; and enter the dilution factor under the **Advanced** tab.
4. Set the camera level to 13 and run the capture script on the NTA software, injecting a fresh batch of sample and entering the temperature of the sample chamber when prompted after each reading.
5. Set the threshold to 4 for the subsequent analysis part, and note the average modal size and particle concentration of the exosome stock from the measurements.

### 5 Characterization of Exosome Purity by Particle:Protein Ratio Determination

1. Measure the protein content of the exosome stock by a bicinchoninic acid (BCA) protein assay kit (see **Table of Materials**) as follows.
  1. Prepare the **defined standards.**
    1. Prepare the standard of the highest concentration (500 µg/mL) by adding 45 µL of BSA stock solution (2 mg/mL – provided in the assay kit) to a microcentrifuge tube, and make it up to 180 µL with PBS.
    2. Fill 8 microcentrifuge tubes with 90 µL of PBS.
    3. Make serial dilutions (factor: 0.5) by taking 90 µL from the highest BSA standard and adding this into the 1st microcentrifuge tube with PBS (mix well), then taking 90 µL from this tube and adding it into the 2^nd^ microcentrifuge tube.
    4. Repeat this until the 7th microcentrifuge tube. The 8th tube will be just PBS (*i.e.,* the blank: 0 µg/mL).
  2. Prepare the **exosome samples** by making 1:2 dilution of samples with PBS in a total volume of 90 µL (45 µL of sample, 45 µL of PBS).
  3. Prepare the BCA working reagent mix.
    1. Calculate the total volume of BCA working reagent mix needed (50 µL per well, in duplicates, including standards).
    2. Mix the individual BCA reagents according to the following ratio: 25 parts reagent A: 24 parts reagent B: 1 part reagent C.
  4. Perform the assay and analysis.
    1. Add 40 µL of each standard and exosome sample prepared above into a well of a 96-well plate (duplicates for each standard and sample).
      NOTE: Since this is a colorimetric assay, proper pipetting technique is crucial to achieve accurate results. Change pipettes after adding each standard/sample replicate into each of the wells
    2. Add 50 µL of the protein assay working reagent mix into each well, and incubate the plate at 37 °C for 30 min.
      NOTE: To minimize deviations between replicates, add the protein assay working reagent into the 1^st^ replicate of a standard/sample, followed by the 2^nd^ replicate of the same sample/standard, before adding it to the 1^st^ replicate of another sample/standard.
    3. Measure the absorbance at 562 nm on the plate reader (see **Table of Materials**).
    4. Plot a standard curve from the absorbance values of the standards, and work out the protein concentration in each sample using the equation of the curve.
2. Calculate the particle:protein ratio by dividing the exosome yield obtained earlier with the protein concentration of the exosome stock measured above.

### 6 Characterization of Exosomal Marker Expression by Flow Cytometry

1. Incubate 40 µL of exosomes (≥1x10^11^ particles/mL) with 10 µL of aldehyde/sulphate latex beads (undiluted from stock) for 15 min at room temperature (RT).
2. Add 5 µL of 100 µM BSA solution (see **Table of Materials**) to the exosome-bead mixture to achieve a 10 mM final concentration and incubate for 15 min at RT.
3. Add 1 mL of PBS and incubate for 75 min at RT in a microcentrifuge tube with mild agitation on a rocking shaker (~150 rpm).
4. Centrifuge the suspension at 580 x g for 5 min at RT and discard the supernatant.
5. Resuspend the pellet with 1 mL of 100 mM glycine solution (see **Table of Materials**) and incubate for 30 min at RT.
6. Centrifuge the suspension for 5 min at 580 x g. Discard the supernatant and resuspend the pellet with 1 mL of 3% FBS/PBS (see **Table of Materials**).
7. Repeat this washing step and resuspend the pellet in 350 µL of 3% FBS/PBS.
8. Divide the suspension into 7 tubes, each containing 50 µL of suspension and incubate them with fluorophore-conjugated anti-CD81, anti-CD9 and anti-CD63 antibodies and their corresponding isotype controls (1:10 dilution), respectively, for 45 min at 4 °C. Keep 1 of the tubes as an unstained control but undergoing the same processing.
9. Add 1 mL of 3% FBS/PBS to each tube, centrifuge for 5 min at 580 x g and discard the supernatant.
10. Resuspend the pellet with 200-400 µL of 3% FBS/PBS and analyze the sample on the flow cytometer (see **Table of Materials**) under the appropriate channels.

### 7 Characterization of Exosome Morphology by Transmission Electron Microscopy (TEM)

1. Fix exosome aqueous dispersions at proper concentrations such as 10^10^ p/mL in fixing solution (see **Table of Materials**) for 15 min.
2. Place the samples on 300 mesh carbon coated copper grids and leave to air dry.
3. Negatively stain the samples with 0.22 µm-filtered aqueous uranyl acetate (see **Table of Materials**) for 4 min followed by two 50% methanol/H_2_O wash (see **Table of Materials**).
4. Air dry the sample.
5. Observe the samples under TEM (see **Table of Materials**). Set the accelerating voltage at 80 kV and the spot size at 2. Use objective aperture with all samples.

### 8 siRNA Encapsulation into Exosomes by Electroporation

1. Pre-chill the electroporation cuvette (see **Table of Materials**) on ice for 30 min before electroporation.
2. Mix 7.0 µg of exosomes (32 µL from 7 x 10^12^ p/mL stock in PBS) with 0.33 µg of siRNA (12 µL from 2 µM stock in RNase-free water) in the microcentrifuge tube. Make up the volume to 150 µL with citric acid buffer (see **Table of Materials**). The exosome to siRNA molar ratio is 1:60 in this case.
3. Transfer the mixture to electroporation cuvette. Cap the cuvette and place it in the cuvette holder of the electroporator (see **Table of Materials**). Rotate the turning wheel 180° clockwise.
  NOTE: The wheel must be turned completely to the locked position, in order for the cuvette to contact the electrodes.
4. Select the desired electroporation program (*e.g.,* X-01, X-05, A-20, T-20, T-30, *etc.*) and start electroporation by pressing the **Start** button. NOTE: A successful pulse is indicated by showing “OK” on the display.
5. Once electroporated, remove the cuvette after turning back the wheel 180° counter-clockwise. Withdraw the sample from the cuvette with the plastic pipette for further processing.

### 9 Removal of Free siRNA Using Size Exclusion Chromatography (SEC)

1. Equilibrate the SEC column (2.9 cm [H] x 1.3 cm [W]; see **Table of Materials**) by passing 3.5 mL of filtered PBS twice.
2. Dissolve 150 μL of electroporated sample in 350 μL of filtered PBS and transfer this to the SEC column to perform the free siRNA removal.
3. Collect the first 500 μL fraction that eluted from the column (F0).
4. Add 500 μL of filtered PBS to the column and collect the next 500 μL fraction (F1).
5. Repeat the above step until a total of 10 x 500 μL fractions (up to F9) is collected. F1 and F2 should contain the siRNA-encapsulated exosomes.
6. Wash the column with filtered PBS (twice, at least) to remove any sample residues.

### 10 *In Vitro* Uptake of siRNA-Loaded Exosomes into PANC-1 Cells

1. Seed PANC-1 cells in 24-well flat-bottom plates (see **Table of Materials**) at a density of 50,000 cells per well 24 h prior to the uptake study and incubate the cells in the incubator (5% CO_2_, 37 °C).
2. Electroporate HEK-293 exosomes (7.0 μg) with Atto655-siRNA (0.33 μg) as per **Step 8**.
3. Purify electroporated exosome as per **Step 9** and resuspend in 100 μL of PBS.
4. Add 50 μL of the electroporated exosomes to PANC-1 cell and incubate at 37 °C and 5% CO_2_ for 4 h.
5. Collect cells after incubation.
6. Wash the cells with 1 mL of sterile PBS and resuspend in 200 μL of PBS in polystyrene round-bottom tube (see **Table of Materials**).
7. Analyze cells by flow cytometer (see **Table of Materials**) with 10,000 events acquired per sample.
  NOTE: Un-electroporated exosome-siRNA mixture samples and untreated cells with filtered PBS were used as controls.

## Representative Results

The physicochemical characterization of exosomes isolated from HEK-293 cells (HEK-293 Exo) are summarized in Table 1. The size measured using nanoparticle tracking analysis (NTA) instrument was 107.0 ± 8.2 nm. Exosome yield from the HEK-293 cells, also analyzed using the NTA instrument, was 6.99 ± 0.22 x 10^12^ p/mL from ~24 mL of CM (obtained from 2 rounds of harvest). Purity of the HEK-293 Exo assessed by calculating the particle-to-protein ratio (P:P) was 8.3 ± 1.7 × 10^10^ p/µg.

**Figure 3:**
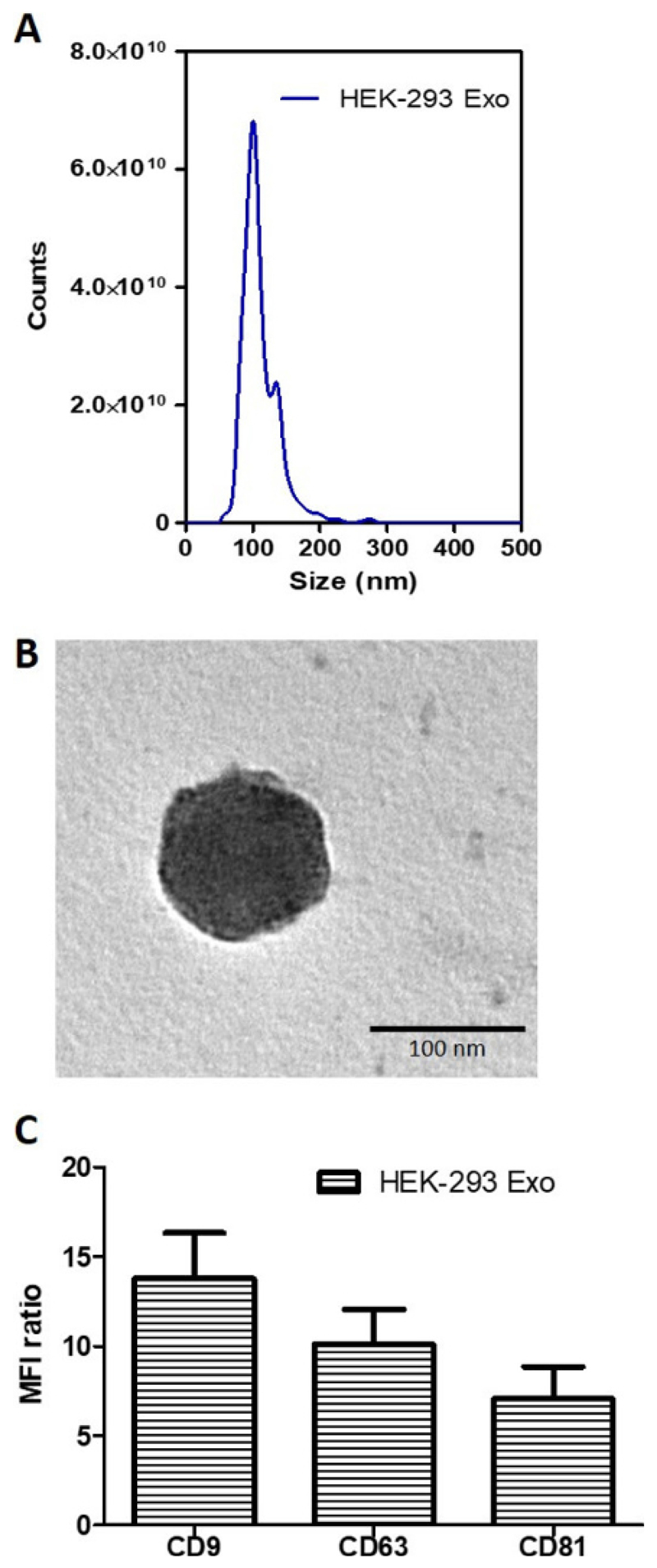
Biochemical and morphology analysis of HEK-293 exosomes. (**A**) Size distribution of HEK-293 exosomes using Nanoparticle Tracking Analysis (NTA). The curve shows a superimposed histogram from 3 different captures at 30 s interval with red areas denoting standard deviation between measurements (n = 3). (**B**) Transmission Electron Microscopy (TEM) images of the naïve HEK-293 exosomes. Scale bar: 100 nm. (**C**) Detection of exosomal markers CD81, CD9 and CD63 using flow cytometry on HEK-293 exosomes. Exosomes were coupled to aldehyde/sulphate latex beads prior to detection. Exosome-beads complex were subsequently stained with fluorophore-conjugated anti-CD81, anti-CD9 and anti-CD63 antibodies. Degree of expression of the markers are expressed as the fold difference in median fluorescence intensity (MFI) values from that of the control (exosome-beads complex stained with the corresponding isotype). Values are expressed as mean ± SD, where n = 3.

The size distribution of isolated HEK-293 Exo is shown in Figure 3A. Morphological analysis using transmission electron microscopy (TEM) showed the HEK-293 Exo were spherical structures slightly above 100 nm in size (Figure 3B). This result agrees with that from NTA measurement (Figure 3A). The isolated HEK-293 Exo were positive for CD81, CD9 and CD63, which are canonical markers used to identify vesicles as exosomes (Figure 3C).

**Figure 4:**
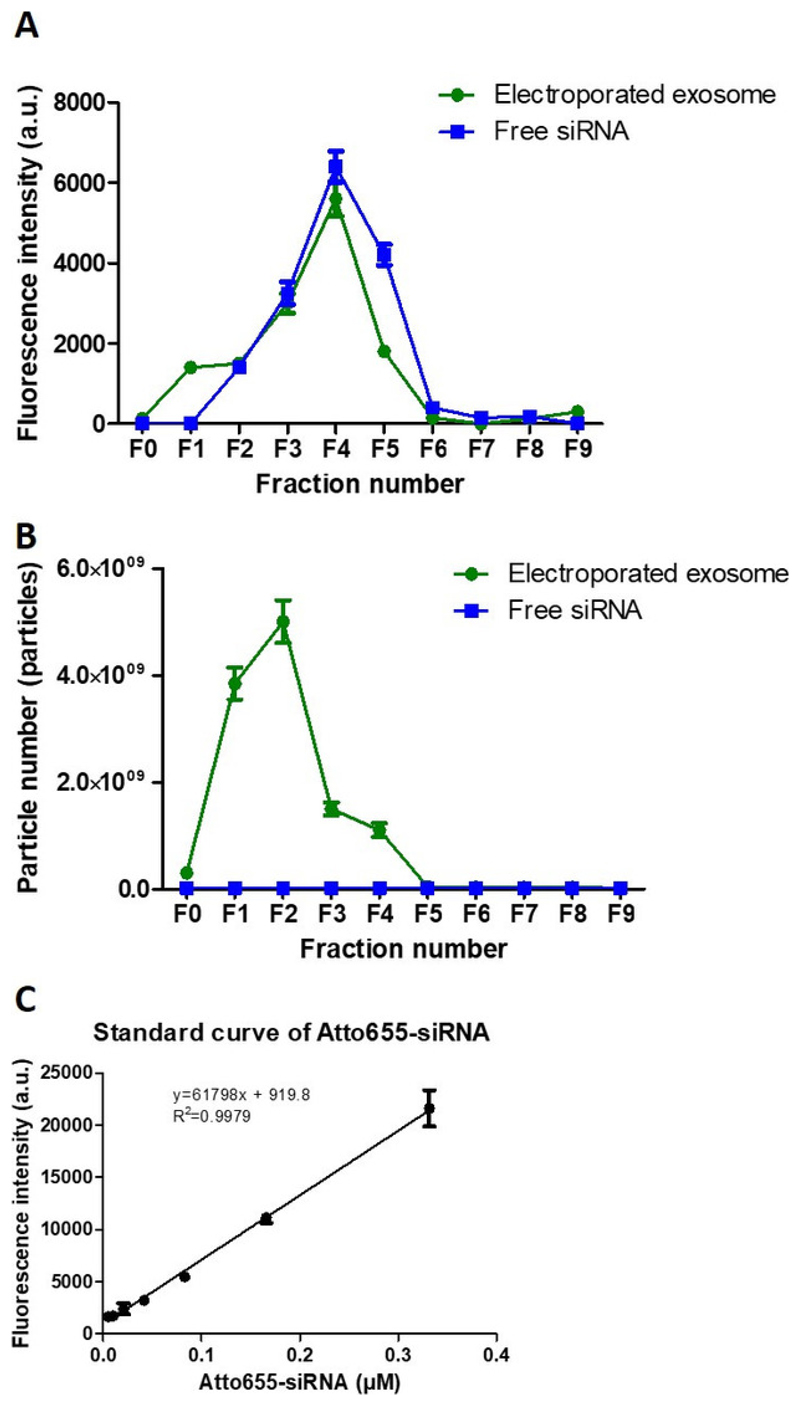
Exosome purification post-electroporation. (**A**) Elution profiles (F0-F9) of Atto655-siRNA and electroporated exosomes using size exclusion chormatography. (**B**) NTA analysis of both Atto655-siRNA and exosome from F0 to F9 using size exclusion chormatography. (**C**) The calibration curve of Atto655 labelled siRNA. Fluorescence intensities were obtained by plate reader at Ex/Em: 640-10/680 nm; Gain 2800. Values are expressed as mean ± SD (n = 3).

For purification of exosomes using size exclusion chromatography (Figure 4), the percentage recovery of exosomes was calculated by dividing the total exosome particle number recovered in the 10 fractions collected (F0-F9) with the initial exosome particle number used, while the percentage recovery of siRNA was calculated by dividing the total fluorescence intensity obtained from F3, F4 and F5 with the total fluorescence intensity obtained from all 10 fractions collected. The recovery of exosome and siRNA post-purification was calculated as 75.0% and 80.4%, respectively. The encapsulation efficiency of siRNA in exosomes was ~10-20%, calculated using the siRNA standard curve established (Figure 4C).

**Figure 5:**
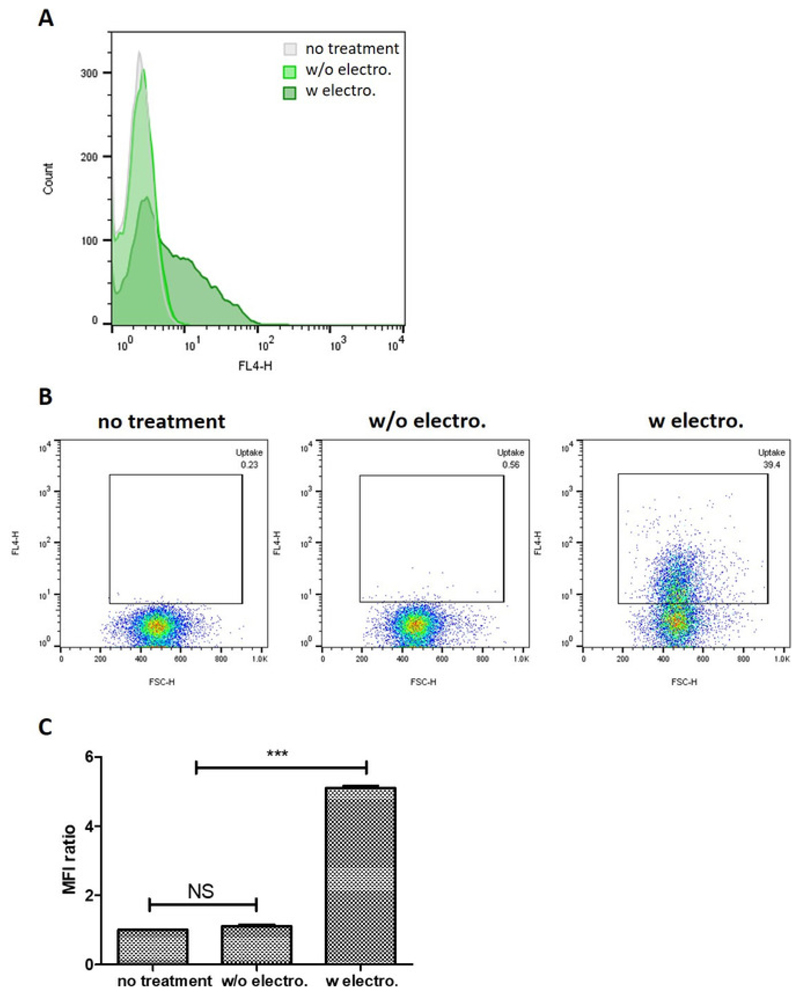
Cellular uptake of siRNA-encapsulated exosomes into PANC-1 cells at 4 h. (**A**) Histograms comparing cellular uptake of unloaded exosomes + siRNA mixture and siRNA-encapsulated exosomes. (**B**) Comparison of the uptake of unloaded exosomes + siRNA mixture at 4h by pseudocolor plot. (**C**) The fold difference in mean fluorescence intensity (MFI) values of the samples tested compared to that of untreated cells. Values are expressed as mean ± SD, where n = 3. *** P < 0.001. NS: not significant. One-way ANOVA was used for statistical analysis.

Qualitative analysis of *in vitro* uptake of exosomes loaded with the fluorescent Atto655-siRNA by flow cytometry showed that PANC-1 cells treated with siRNA-encapsulated exosomes recorded the largest shift in fluorescence signal (Figure 5A). PANC-1 cells treated with siRNA-encapsulated exosomes recorded a higher percentage of population positive for the Atto655 signal (39.4%) compared to that treated with unloaded exosomes and siRNA mixture (0.56%), which corroborated the observation above (Figure 5B). The degree of cellular uptake of siRNA (expressed as the fold difference in mean fluorescence intensity (MFI) values from that of untreated cells) was also observed to be significantly higher in PANC-1 cells treated with siRNA-encapsulated exosomes (MFI fold difference = 5.1) compared to that treated with the exosome-siRNA mixture (MFI fold difference = 1.1) (Figure 5C). These observations demonstrated that the siRNA-encapsulated exosomes were internalized by the PANC-1 cells and that they effectively delivered the siRNA intracellularly.

## Discussion

Obtaining a decent exosome yield from cultured cells, which are enough for several rounds of *in vitro* or *in vivo* studies, is still a challenge. According to the manufacturer, the bioreactor flasks were intended for production of antibodies and proteins with high yield from culture of various immortalized cell lines. This allows the cells to continuously enrich the culture medium with the desired product, resulting in a concentrated conditioned medium (CM) in the cell-compartment. Theoretically, the same concept would be beneficial in exosome production from various cell lines, and indeed culturing these cells in the bioreactor flasks was demonstrated to significantly increase the exosome yield^40^. The large medium reservoir continuously supplies nutrients to and removes wastes from the cell compartment through a 10 kDa semi-permeable membrane, allowing prolonged culture without requiring a large volume of medium to be in contact with the cells, or regular flasks changing, which can ultimately save the overall cost and labor of high-scale exosome production^40^. It was also demonstrated that the morphology, phenotype as well as the immunomodulatory functions of exosomes isolated cells long-term bioreactor flasks cultures are similar to that sourced from cells cultured in regular 75 cm^2^ flasks^40^. Culture of other immortalized cell lines as exosome sources in the bioreactor flask would therefore help increase their exosome yield while maintaining their integrity and function. This form of culture is however not applicable to primary cells with limited division cycles, and those that cannot be cultured in high density.

Since harvest of the CM is done weekly, and the cells in culture were never passaged, it can be assumed that the cells in the bioreactor flask are not growing in a monolayer like the regular cell culture. They are most likely to form clusters with necrotic centers, or simply detach from the surface and die when the cells are too confluent for a monolayer. Visual inspection of the cell compartment of the bioreactor flask is not possible to confirm this assumption, but is reflected by the large number of dead cells obtained during the CM harvesting. Regular removal of poorly adherent and non-viable cells from the bioreactor flask can prevent the build-up of materials on the semi-permeable membrane that can adversely affect the exchange of gas, nutrients and waste between the cell compartment and the medium reservoir, thus allowing prolonged culture in the bioreactor flasks for >6 months^40^. In this context, this non-regularity of cell growth in the bioreactor flasks is ideal as we speculate that it mimics the actual condition of tumor growth *in vivo* more closely than the conventional monolayer cell culture, and it is hoped that the exosomes produced by the cancer cells in the bioreactor flask would be more similar to that secreted by tumors *in vivo*. This would be particularly beneficial in studies looking into the role of tumor-derived exosomes in the progression of the tumor pathology. Tumor-derived exosomes have been reported to intrinsically and preferentially home to their tissue of origin^32^, therefore having exosomes produced in a system mimicking their *in vivo* production would also be desirable in studies looking at exploring the passive targeting ability of exosomes as drug nanocarriers.

The P:P ratio was reported as a parameter to assess the purity of isolated exosomes from contaminating proteins from the culture medium of physiological fluids from which exosomes were sourced from^41^. The P:P ratio of 8.3 ± 1.7 × 10^10^ p/µg obtained in this study falls within the high purity range proposed in the study. This ratio highlights the danger of using protein concentration to express the yield or dose of exosomes isolated or used in downstream studies respectively, as this does not reflect the true amount of exosomes available in the sample given the problem of protein contamination during isolation. NTA *via* instruments such as NanoSight, which measures the concentration of exosomes in terms of particle number, is a more sensible and accurate way of quantifying exosomes.

Highly accurate weighing during the preparation of the 25% sucrose solution in deuterium oxide is crucial as this method is a density-based isolation. Exosomes have a rather narrow range of flotation density in sucrose solution so accurate preparation of the sucrose cushion will reduce contamination of non-exosomal vesicles such as apoptotic bodies or Golgi-derived vesicles during isolation^42^. It is advised not to keep leftover sucrose solution and using it even after one day so as to avoid risk of factors that can alter its density such as loss or addition of water in the solution by either evaporation or condensation of air in the tube. Use of a swing-out rotor is also essential during centrifugation onto the sucrose cushion to allow even migration of exosomes from the CM to the sucrose solution.

Withdrawing the sucrose solution post-centrifugation is also a delicate step, and it involves finding a compromise between maximizing the amount of exosomes recovered, and not too much that protein from culture medium is introduced to the exosome sample withdrawn. The interface between the sucrose solution and the condition medium is where proteins from the culture medium would collect post-centrifugation, and can usually be seen as a dark brown ring that sits on the interface. In our hands, withdrawing 2 mL of the sucrose cushion from the initial 3 mL added is the optimum volume that agrees with the compromise mentioned above. The volumes described in this protocol are for the specific rotors used; therefore, it is advised to optimize the volume of sucrose to be withdrawn when scaling up or down the volumes for the types of rotors available in different facilities. It is also important to avoid the area right at the center of the bottom of the tube when withdrawing the sucrose, as this is where particles of higher density than sucrose will sediment and can usually be seen as an off-white pellet.

The washing step with a relatively large amount of PBS helps to further reduce the degree of protein contamination during exosome isolation^41^. This step is also essential in removing excess sucrose from the exosomes so as to avoid osmotic damage to the exosomes themselves or the biomolecules within the exosomal lumen, as well as reducing the risk of bacterial and/or fungal growth in the exosome stock. Preparing the sucrose solution in deuterium oxide rather than water helps to reduce the amount of sucrose needed to achieve the exosome flotation density for isolation, hence reducing the risk of both osmotic damage and microbial contamination. After the first centrifugation onto the sucrose cushion, the exosome-containing sucrose layer withdrawn and added to the PBS can be stored at 4 °C and processed the following day if faced with time constraints.

To the best of our knowledge, the exosome/siRNA molar ratio is an important factor in determining the efficiency of electroporation. In this protocol, we used 1:60 as the exosome to siRNA molar ratio. As the encapsulation ability of different types of exosomes are different, we strongly suggest this to be optimized on a case-by-case basis. However, the encapsulation efficiency proposed herein can always be a parameter for selecting the optimal electroporation conditions.

In addition, aggregation of siRNA is believed to be one of the most common problem in electroporation. It is proven that electroporation can induce strong aggregation of siRNA, making it even harder to enter exosomes. siRNA aggregations are often mistakenly interpreted as encapsulation of siRNA into exosome therefore proper controls were used in this study as the formation of siRNA aggregates is unavoidable during electroporation^28^. The percentage encapsulation efficiency of our purification method was calculated by using normalized values to minimize the influence from other sources such as background noise, exosome and siRNA aggregations that would affect the data reliability. Based on our findings, there was negligible siRNA aggregations observed in the control sample *i.e.,* using electroporated and un-electroporated siRNA.

This protocol has successfully demonstrated the encapsulation of siRNA into exosomes and their subsequent intracellular delivery of the siRNA to cancer cells *in vitro*. Therefore, various types of exosomes from different cell lines can be isolated and characterized using the proposed protocol, and subsequently loaded with various therapeutic siRNA for different types of oncogenic targets over-expressed in different cancers. An interesting application would be to explore the siRNA delivery and uptake efficiency using various permutations of exosome source-target cell pair *in vitro*. This can then be translated to animal models to assess the efficiency of both the delivery and therapeutic efficiency of siRNA-encapsulated exosomes *in vivo.*

## Figures and Tables

**Figure 1 F1:**
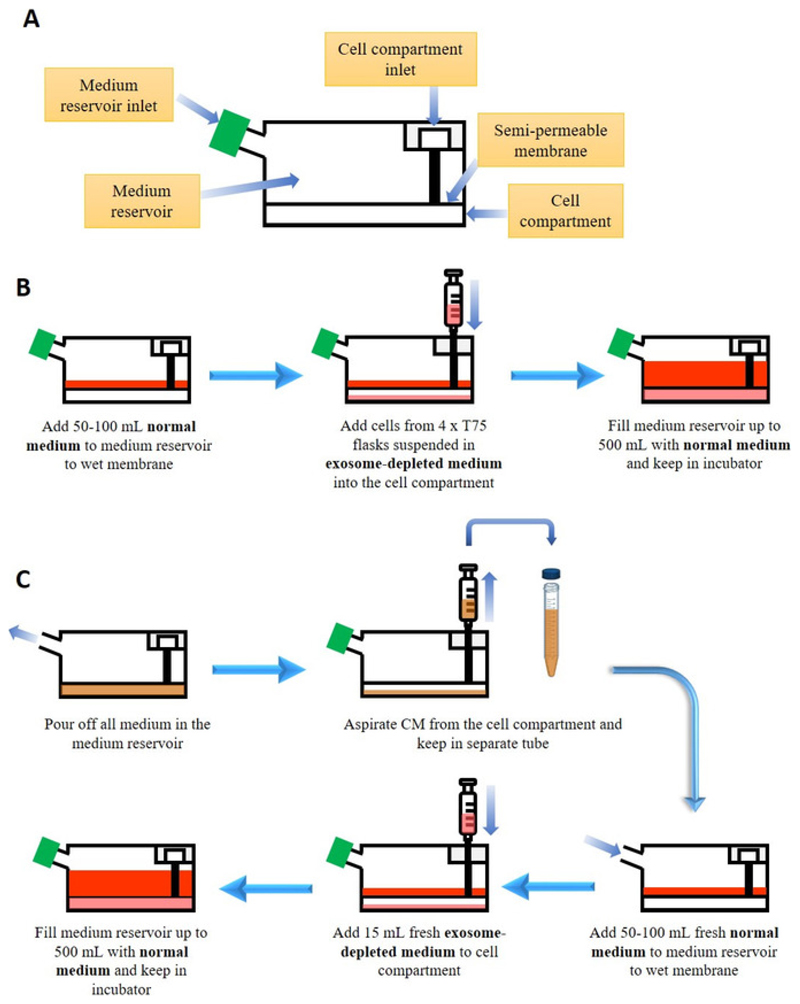
Culture of cells in bioreactor flask for exosome production. (**A**) Simplified anatomy of the bioreactor flask. (**B**) Starting culture in the bioreactor flask. See **Table of Materials** for the composition of normal and exosome-depleted medium (**C**) Harvesting conditioned medium (CM) and maintenance of culture in the bioreactor flask.

**Figure 2 F2:**
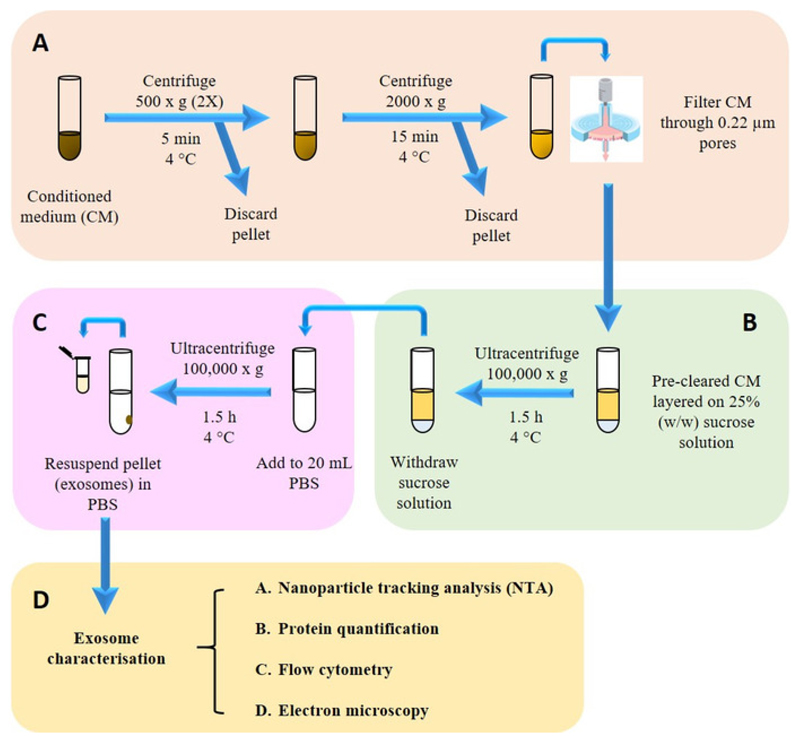
Isolation and characterization of exosomes. (**A**) Pre-clearing harvested conditioned medium (CM) from dead cells and cell debris. (**B**) Isolating exosomes from CM onto sucrose cushion. (C) Washing step to remove sucrose and contaminating proteins. (**D**) Isolated exosomes were then subjected to physicochemical, biochemical and morphological characterization.

**Table 1:** Physicochemical characterization of exosomes.

Exosome	Size^1^,^2^ (nm)	Yield^1^,^2^,^3^ (p/mL)	[Protein]^2^,^4^ (µg/mL)	Particle-to-protein (P:P) ratio^5^ (p/µg)
HEK-293	107.0 ± 8.2	6.99 ± 0.22 x 10^12^	84.3 ± 9.8	8.3 ± 1.7 x 10^10^

1 Measured using nanoparticle tracking analysis (NTA instrument)

2 Values are expressed as mean ± SD, where n=3

3 Yield was obtained by cell-conditioned medium pooled from 2 rounds of harvesting from bioreactor flasks (~24 mL)

4 Measured using a protein assay kit

5 Value obtained by using formula: P:P ratio = Yield / [Protein]
